# Patient safety culture in nursing homes – a cross-sectional study among nurses and nursing aides caring for residents with diabetes

**DOI:** 10.1186/s12912-018-0305-z

**Published:** 2018-08-07

**Authors:** Irit Titlestad, Anne Haugstvedt, Jannicke Igland, Marit Graue

**Affiliations:** 1 Faculty of Health and Social Sciences, Western Norway University of Applied Sciences, Postbox 7030, N-5020 Bergen, Norway; 2 Kleppestø Nursing Home, Askøy Municipality, Bergen, Norway; 3 Department of Global Public Health and Primary Care, University of Bergen, Bergen, Norway

**Keywords:** Nursing home, Nursing personnel, Patient safety culture, Diabetes

## Abstract

**Background:**

Due to the high morbidity and disability level among diabetes patients in nursing homes, the conditions for caregivers are exceedingly complex and challenging. The patient safety culture in nursing homes should be evaluated in order to improve patient safety and the quality of care. Thus, the aim of this study was to examine the perceptions of patient safety culture of nursing personnel in nursing homes, and its associations with the participants’ (i) profession, (ii) education, (iii) specific knowledge related to their own residents with diabetes, and (iv) familiarity with clinical diabetes guidelines for older people.

**Methods:**

Cross-sectional survey design. The study included 89 nursing home personnel (38 registered nurses and 51 nurse aides), 25 (28%) with advanced education, at two nursing homes. We collected self-reported questionnaire data on age, profession, education and work experience, diabetes knowledge and familiarity with diabetes guidelines. In addition, we applied the Nursing Home Survey on Patient Safety Culture instrument, with 42 items and 12 dimensions.

**Results:**

In general, those with advanced education scored higher in all patient safety culture dimensions than those without, however statistically significant only for the dimensions “teamwork” (mean score 81.7 and 67.7, *p* = 0.042) and “overall perceptions of resident safety” (mean score 90.0 and 74.3, *p* = 0.016). Nursing personnel who were familiar with diabetes guidelines for older people had more positive perceptions in key areas of patient safety culture, than those without familiarity with the guidelines.

**Conclusions:**

The findings from this study show that advanced education and familiarity with current diabetes guidelines was related to adequate evaluations on essential areas of patient safety culture in nursing homes.

## Background

The increasing prevalence of diabetes worldwide as well as the increasing number of older individuals in many societies will lead to an expected rise in the number of older individuals with diabetes in nursing homes in the years to come [1–3]. A systematic review has shown a variation in the prevalence of diabetes in nursing homes from 8 to 53%, with a mean prevalence of 18.5% [4]. Studies in Norway have indicated the prevalence of diabetes in Norwegian nursing homes to be 15–17% [5, 6]. Nursing home residents with diabetes have a higher burden of comorbidity and are considered to be a vulnerable and neglected group of patients who suffer from a high level of both physical and cognitive impairment [3]. Thus, residents with diabetes are a complex and challenging care group in nursing homes [2]. Nursing personnel must handle an extensive list of medications, treatments and care deficiencies that are both complicated and time-consuming. National clinical diabetes guidelines are intended to support and give concrete recommendations in relation to diabetes care, to ensure patient safety and minimize adverse events and variations in clinical practice [7, 8]. In Norway, the municipalities are responsible for providing care for inhabitants in need, and almost all nursing homes are public. The main groups of nursing personnel in the nursing homes are registered nurses (with education on university/university college level), nursing aides (with education on upper secondary school level) and assistants without any formal education. Nurses and nursing aides authorized to administer medication, are primarily the nursing personnel who need the knowledge and expertise to secure high quality care for residents with diabetes.

Patient safety is a topic that internationally has received increased professional and political attention in recent years [9, 10]. It is recommended to evaluate the patient safety culture (PSC) in health care institutions to subsequently improve patient safety and quality of care [11–16]. Research has shown that there is a relationship between weak PSC and clinical outcomes, where a higher perception of PSC is associated with better patient outcomes [17, 18]. The health providers’ knowledge and expertise, and familiarity with national clinical recommendations in relation to treatment and care within a specific clinical field, might influence PSC and thus partially explain the observed associations between PSC and clinical outcomes. Clinical guidelines intends to ensure the best possible patients safety by serving as a tool for health professionals and patients to take proper and responsible decisions, and also by contributing to less adverse variations in practice and improved quality of health care [7]. However, studies have shown that diabetes guidelines in nursing homes are not followed closely enough and that the quality of care could be improved by better use of the guidelines [4].

In a previous study, we have shown insufficient diabetes knowledge among nurses, nursing aides and nursing assistants in nursing homes in Norway [19]. Although the nurses reported better diabetes knowledge than the nursing aides and nursing assistants, all groups lacked knowledge on important topics related to diabetes treatment and care. In that study we did, however, not study the participants familiarity with clinical diabetes guidelines or if the lack of knowledge was related to PSC. Thus, in this study, we will explore possible associations between PSC and nursing personnel’s knowledge and expertise, and familiarity with clinical diabetes guidelines in relation to diabetes treatment and care for older people.

## Methods

### Aim

We aimed to examine the perception of PSC among nursing home personnel, and its associations with the participants’ (i) profession (registered nurses vs. nursing aides), (ii) education (with or without advanced education), (iii) specific knowledge related to their own residents with diabetes, and (iv) familiarity with clinical diabetes guidelines for older people.

### Design and study population

We conducted the study from August to September 2015. We invited registered nurses and nursing aides at two nursing homes in Western Norway to participate in this cross-sectional study. The nursing homes had respectively 123 and 107 residents divided on long-term care units (for about 80% of the residents), short-term units and rehabilitation units. Of the respectively 89 and 93 registered nurses and nursing aides working in the two nursing homes, 31 (35%) and 36 (39%) was registered nurses. Thus, nursing aides constituted the largest personnel group in the nursing homes.

### Measures

#### Demographic variables

We collected data on age (“18–29 years”, “30–39 years”, “40–49 years”, “50–59 years” or “60 years or older”); profession (“registered nurse”, “nursing aides” or “other”), continuing education (with advanced education or without) and time since completing education (“less than 5 years”, “5–10 years”, “11–15 years”, “16–20 years” or “more than 20 years”). The nursing personnel was not asked specifically what their advanced education included, but for nurses advanced education typically included geriatric or palliative education while for nursing aides was geriatric or psychiatric courses. In addition, nursing home work experience (“1–5 years”, “6–10 years”, “11–15 years”, “16–20 years” or “21 years or more”), status of employment (“permanent” or “temporary”), (“full” or “part-time”) and work shift (“day, evening and night”, “day and evening”, “only night” or “others”) were assessed.

### Specific knowledge related to their own residents with diabetes

The questionnaire included questions about the participants’ familiarity with clinical diabetes guidelines for older people (“yes”, “no”), if they administered insulin (“yes”, “no”, “don’t know”) and whether they knew if any of their residents were on glucose lowering drugs which could cause hypoglycaemia (“yes”, “no”,“don’t know”). In addition, specific knowledge related to their own residents with diabetes included questions about glycaemic treatment goals, routines for blood glucose monitoring and risk assessment in relation to hypoglycaemia [20].

### Nursing home survey on patient safety culture

We used the Nursing Home Survey on Patient Safety Culture (NHSPSC) to collect data on the participants’ perceptions of PSC [21]. The NHSPSC was developed in 2008 by the Agency for Healthcare Research and Quality to measure PSC among nursing homes personnel [12, 22], and has been translated into Norwegian by Cappelen et al. [23]. The NHSPSC is composed of a total of 42 items divided into 12 dimensions with three to four items in each dimension, and items can be either positively and negatively worded [21]. The 12 dimensions are: the respondents’ perception of “teamwork”, “staffing”, “compliance with procedures”, “training and skills”, “response to mistakes”, “handoffs (information concerning patient transition)”, “feedback and communication about incidents”, “communication openness”, “supervisor expectations and actions promoting resident safety”, “overall resident safety”, “management support for resident safety” and “organizational learning”. The response alternatives on these items are either “strongly disagree”, “disagree”, “neither”, “agree”, “strongly agree”, and “not applicable/do not know” or “never”, “rarely”, “sometimes”, “most of the time” and “always”. The answers “agree” and “strongly agree” were defined as a positive response on positively worded items, while “disagree” and “strongly disagree” were defined as positive responses on negatively worded items. In addition, the instrument contains two single questions about (i) the respondents’ general opinion about the PSC in the nursing home and (ii) the number of reported adverse events during the past year [21]. A respondents’ individual score for each NHSPSC dimension was estimated by calculating the number of positive responses for each dimension, divided by the number of items in the dimension, and multiplying by 100. In cases where a participant did not answer one or more items (missing), the dimension score was calculated according to the actual number of items each individual had responded to. Individual dimension scores are ranged from 0 to 100, where higher scores indicate a more positive response [21]. The method used in the present study to calculate individual scores have been used in previous studies [14] and results in mean scores which will be equal to the score for the total study population according to the instructions in the manual [21]. Mean scores in the present study can therefore be compared with the results from studies in which scores are calculated at the institutional level [24]. A validation study of the Norwegian version of the NHSPSC by Cappelen et al. [23], indicated satisfactory internal consistency with Cronbach’s alpha for the different dimensions ranging from 0.55 to 0.90. However, results from confirmatory factor analyses gave improved model fit after merging of the three last dimensions (“organizational learning”, “overall perceptions of patient safety” and “management support for patient safety”), resulting in 10 dimensions instead of 12 [23]. In our study, Cronbach’s alpha ranged from 0.50 to 0.73 for the 12 dimensions. We also made a combination of the three last dimensions in accordance with Cappelen et al. [23] in order to explore if this changed any of our results for comparison of PSC between groups. Reliability analysis of the new combined dimension proved to have a good Cronbach’s alpha value (0.77), while each dimension separately was found to have a Cronbach’s alpha score lower than 0.60.

### Data analysis

The study population was characterized using descriptive statistics (counts and percentages) (Table 1). Association between categorical variables were tested using chi-square tests. The associations between NHSPSC dimension scores and demographic- and diabetes-related questions were analysed by independent sample t-tests, and reported as mean, standard deviation and *P*-values. In addition to analyses for each of the 12 original dimensions, we also did analyses for the combination of the three last dimensions according to the dimension suggested by Cappelen et al. [23]. The SPSS statistical program package Version 21; SPSS Inc.Chicago, IL, USA was used, and statistical significance was defined as *P* < 0.05.

**Table 1:** Characteristics of the study population (*N* = 89)

	*n* (%)
Profession group	
Registered Nurses	38 (42.7)
Nurse Aides	51 (57.3)
Nursing home	
Nursing home 1	50 (56.2)
Nursing home 2	39 (43.8)
Age (years)	
18–29	5 (5.6)
30–39	14 (15.7)
40–49	29 (32.6)
50–59	28 (31.5)
≥ 60	12 (13.5)
Missing	1 (1.1)
Time since completing education (years)	
< 5	13 (14.6)
5–10	22 (24.7)
11–15	19 (21.4)
16–20	13 (14.6)
> 20	22 (24.7)
Advanced education	
Yes	25 (28.1)
No	62 (69.7)
Missing	2 (2.2)
Work experience from nursing home (years)	
1–5	5 (5.6)
6–10	24 (27.0)
11–15	17 (19.1)
16–20	18 (20.2)
≥ 21	25 (28.1)
Conditions of employment	
Permanent	88 (98.9)
Temporary	1 (1.1)
Job content	
Full	52 (58.4)
Part-time	36 (40.5)
Missing	1 (1.1)
Work shift	
Day, evening and night	7 (7.9)
Day and evening	76 (85.4)
Only night	1 (1.1)
Others	5 (5.6)

### Ethics

The head nurses in the two participating nursing homes participated in the planning phase of the study. The Norwegian Centre for Research Data approved the study (Ref No: 2015/43349). All participants gave informed consent. Data collection took place at specially allotted rooms outside the participants’ work unit in the nursing home.

## Results

Of the 182 eligible nursing personnel from the two included nursing homes 89 individuals completed the study questionnaire. This gave response rates in the two nursing homes of 56 and 44%, respectively. Of the 89 responders, 38 (43%) were registered nurses and 51 (57%) were nursing aides (Table 1). Furthermore, 25 (28%) of the 89 participants had any kind of advanced education with a non-significant difference between professions (36.8% among nurses and 22.4% among nursing aides, *p* = 0.14). Totally 49 (55%) of the participants reported that they were familiar with the Norwegian diabetes guidelines for older people with diabetes in nursing homes, with a slightly higher proportion among nurses, but not significant (60.5% among nurses and 51.0% among nursing aides, *p* = 0.37). Advanced education in itself was not significantly associated with familiarity to the diabetes guidelines (44% among nursing personnel with advanced education and 58.1% among nursing personnel without advanced education reported familiarity to the diabetes guidelines, *p* = 0.23). In total 58 (65%) of the participants answered “yes” on the question whether they knew if any of their residents were on glucose lowering drugs which could cause hypoglycaemia, and 73 (82%) reported that they administered insulin with a significantly higher proportion among nurses compared to nursing aides (92.1% versus 74.5%, *p* = 0.03).

Nurses had significantly higher score on the PSC dimension “communication openness” compared to nursing aides (mean 71.0 versus 47.4, *p* = 0.004). For the other dimensions, including the new combined dimension, no significant differences between nurses and nursing aides were identified (results not shown in tables). We identified that nursing personnel with advanced education reported significantly more positive perceptions of the dimensions “teamwork” and “overall perceptions of resident safety” compared to those without advanced education, whereas the dimension “management support for resident safety” was borderline significant (*p* = 0.051) (Table 2). The difference was also significant for the new combined dimension. The ones with advanced education also responded more positively to all the other patient safety dimensions, but the differences were not statistically significant. We found a borderline significant difference on perception of “compliance with procedures” between the nursing personnel who did not know if their patients’ blood glucose lowering drugs could cause hypoglycaemia and those who did have such knowledge, with highest score among those without knowledge (mean 67.9 versus 52.6, *P* = 0.050). For the other dimensions, including the new combined dimension, there were no significant differences between nursing personnel with and without knowledge of patients on glucose lowering drugs. However, the differences between the groups in four of the dimensions were higher than 5–10 points; “staffing” (mean 45.4, versus 38.4 *p* = 0.183), “training and skills” (mean 55.6 versus 46.8, *p* = 0.303), “nonpunitive response to mistakes” (mean 73.8 versus 67.1, *p* = 0.387) and “overall perceptions of resident safety” (mean 87.7 versus 75.7, *p* = 0.079).

**Table 2:** Nursing personnel’s with and without advanced education mean patient safety culture score for the 12 dimensions in the NHSPSC^a^ (*N* = 87)

	With advanced education Mean (SD) *n* = 25	Without advanced education Mean (SD) *n* = 62	*P* Value ^b^
Positive responses to			
1. Teamwork	81.7 (25.7)	67.7 (33.8)	*0.042* ^b^
2. Staffing	43.7 (16.4)	37.2 (25.7)	0.169
3. Compliance with procedures	62.5 (33.1)	54.3 (35.8)	0.319
4. Training and skills	54.0 (33.1)	44.6 (38.1)	0.258
5. Nonpunitive response to mistakes	69.0 (30.0)	68.5 (34.2)	0.952
6. Handoffs (information concerning patient transition)	54.3 (31.1)	50.7 (34.9)	0.634
7. Feedback and communication about incidents	86.0 (28.9)	82.3 (26.5)	0.579
8. Communication openness	63.3 (34.7)	55.6 (39.9)	0.375
9. Supervisor expectations and actions promoting resident safety	85.3 (32.0)	73.2 (34.9)	0.127
10. Overall perceptions of resident safety	90.0 (23.6)	74.3 (32.9)	*0.016* ^b^
11. Management support for resident safety	54.3 (32.3)	37.4 (39.9)	0.051
12. Organizational learning	64.3 (30.7)	50.7 (36.1)	0.082
Combined dimension (10, 11 and 12)	69.6 (23.5)	54.1 (31.6)	*0.016* ^b^

^a^Score for each person is calculates as percent positive responses within each dimension. Score for each dimension may vary between 0 and 100%. Higher scores indicate more positive responses

^b^Significantly different t-test at *P* < 0.05

Our study indicated significant associations between the participants’ perceived familiarity with the Norwegian diabetes guidelines for older nursing homes residents and their perceptions of several NHSPSC dimensions (Table 3). Those who claimed to be familiar with the guidelines on average had more positive scores on a larger proportion of questions within the dimensions “handoffs” (60.9 versus 42.9, *P* = 0.013), “supervisor expectations and actions promoting resident safety” (85.4 versus 67.5, *P* = 0.015), “management support for resident safety” (54.3 versus 30.0, *P* < 0.003) and “organizational learning” (63.4 versus 44.9, *P* = 0.014). The total patient safety culture scores were 11% higher among those who were familiar with the guidelines compared to those who were not (*P* = 0.021). Those who were familiar with the guidelines also scored significantly higher for the new combined dimension (*p* = 0.003).

**Table 3:** Nursing personnel’s mean patient safety culture score for the 12 dimensions in the NHSPSC in relation to familiarity to the Norwegian clinical diabetes guidelines for elderly nursing home residents^a^ (*N* = 89)

	Nursing personnel who reported familiarity to the Norwegian clinical diabetes guidelines Mean (SD) *n* = 49	Nursing personnel who reported no familiarity to the Norwegian clinical diabetes guidelines Mean (SD) *n* = 40	*P* Value ^b^
Positive responses to:			
Teamwork	77.2 (26.2)	65.2 (36.8)	0.088
Staffing	39.8 (25.8)	38.8 (20.9)	0.833
Compliance with procedures	56.9 (35.0)	56.7 (34.8)	0.970
Training and skills	53.1 (39.6)	42.9 (33.7)	0.196
Nonpunitive response to mistakes	73.1 (34.1)	62.3 (32.1)	0.127
Handoffs (information concerning patient transition)	60.9 (33.2)	42.9 (33.0)	*0.013* ^b^
Feedback and communication about incidents	87.6 (24.1)	78.1 (29.5)	0.107
Communication openness	58.5 (36.8)	56.7 (40.1)	0.824
Supervisor expectations and actions promoting resident safety	85.4 (29.9)	67.5 (36.6)	*0.015* ^b^
Overall perceptions of resident safety	84.0 (29.0)	73.8 (32.7)	0.126
Management support for resident safety	54.3 (37.1)	30.0 (36.6)	*0.003* ^b^
Organizational learning	63.4 (31.4)	44.9 (36.1)	*0.014* ^b^
Combined dimension (10, 11 and 12)	67.7 (27.5)	49.0 (30.1)	*0.003* ^b^

^a^Score for each person is calculates as percent positive responses within each dimension. Score for each dimension may vary between 0 and 100%. Higher scores indicate more positive responses

^b^Significantly different t-test at *P* < 0.05

## Discussion

To our knowledge, this is the first study examining PSC among nursing home personnel and its associations with the participants’ familiarity with diabetes guidelines for older people. We found that personnel with advanced education and familiarity with guidelines had a more positive perception in key areas of PSC. Moreover, safety culture perceptions differed significantly between registered nurses and nursing aides relative to the dimension, “communication openness” in the NHSPSC. It might be that registered nurses are more able to speak up about problems and at the same time, their suggestions might be more valued than those of nursing aides. Castle [25] also discusses this matter, related to differences in perception of PSC among professions. In that study, higher perception of PSC was associated with higher registered nurse staffing levels. As nursing aides represent a large part of the staffing in nursing homes in many countries, the low positive scoring on “communication openness” among nursing aides in our study (47.7%) may arouse some concerns. Communication between nursing personnel is a particularly important concern for quality of care and may therefore influence on patient safety. Other researchers have also suggested that failure of communication between personnel within the health system might be associated with individual and structural factors and lack of knowledge and skills [26] putting patient safety and patient outcomes in danger [11]. Sinclair [3] points out that there are a number of important barriers to improving diabetes care in nursing homes. One obstacle is inadequate training of nursing personnel in basic diabetes care, and lack of resources for such training. In our study, 35% of the participants’ lack knowledge about which of the glucose lowering medications that can give hypoglycaemia. Another important barrier for improving care is miscommunication between nursing personnel, which occur due to the lack of clear professional boundaries and responsibilities, as well as the lack of national standards for diabetes care in nursing homes [3]. According to Danielsson et al. [27], efforts for improving PSC have to take into account the profession groups’ values, norms, and assumptions related to patient safety.

This study also showed that advanced education was associated with better perception of PSC. Even though there were statistically significant differences only in three dimensions, the difference between the groups in nine of the dimensions was higher than 5–10 points. For a variety of questionnaire-based scales, changes between 5 and 10% (or five to 10 points on a 100-point scale) might be considered large enough to be noticed by people and thus regarded as important [28]. We do therefore think that also the non-significant differences are noteworthy. In our sample, we did not have the power to elaborate this further; however, further research might examine more in detail whether advanced education, and the duration of training in itself, might play a role for patient safety. Nursing home patients with diabetes usually have several additional chronic conditions, and therefore treatment and care for this group of patients is more challenging [3, 29]. The higher the competence among nursing personnel are, the more knowledge, skills and abilities to handle different situations, both in terms of direct patient contact, cooperation between nursing personnel, and between different professions. Nursing personnel with higher competence may take a more comprehensive approach to nursing and patient safety. Findings from a large survey that was carried out among nurses working in municipal health care in Norway showed that approximately 70% of the participants reported that the need for more nurses with advanced education or a master’s degree is increasing [26]. This relates to the increasing number of older people with higher burden of comorbidity and a range of chronic conditions requiring demanding medical treatment and procedures [2]. Findings from the study of Agarwal et al. [30] showed that nursing personnel reported a need for more training and updating of knowledge on various topics related to diabetes.

Findings concerning whether nursing personnel had knowledge of which patients were using blood glucose lowering drugs that can cause hypoglycaemia show that those who claimed to lack this knowledge scored borderline significantly higher in one dimension (“compliance with procedures”) than those who claimed to have the knowledge. They scored higher in six other dimensions as well, but without statistically significant differences. However, the differences between the groups in four of the dimensions were higher than 5–10 points and were therefore perceived as being important [28]. Knowledge of which patients are using these medications appears to be important for the perception of patient safety because hypoglycaemia among frail elderly may lead to worsening of chronic diseases, cognitive impairment, increased morbidity and mortality [31]. In a way, it makes sense to assume that those with knowledge of which patients were using blood glucose lowering drugs that can cause hypoglycaemia also would report higher scores on perception of PSC. On the other hand, they might not have had the knowledge about how to handle side effects, such as hypoglycaemia. Thus, the knowledge of which patients who are on blood glucose lowering drugs might not necessarily play a role related to differences in perception of PSC. The somewhat unexpected result led us to consider that these findings were influenced by other variables that we did not examine in this study.

We found associations between some areas of PSC, and whether or not nursing personnel reported familiarity with diabetes guidelines for older people. Kuehn [32] points to studies suggesting that guidelines can help ensure quick application of new knowledge in practice, reducing undesirable variations and improving safety and quality. However, in routine practice not all patients may actually receive the most appropriate treatment based on research evidence [33]. The fact that only 55% of the participants in this study reported familiarity with the diabetes guidelines for older nursing home residents can raise questions about where in the system dissemination of new and relevant knowledge fails, and why. Promoting the implementation of clinical guidelines in Norwegian nursing homes and not at least appropriate use of guidelines can lead to awareness among nursing personnel of the need to take informed decisions [34], which can enhance patient safety [7].

### Limitations

The sample was a nonprobability sample, which can be exposed to bias [35]. In addition, the sample was small, with a relatively low response rate, which made it difficult to identify significant differences between groups. Moreover, we did not have access to background information on those who chose not to participate (except for their profession) and whether they differed from those who participated, for example in relation to gender, age and their educational level. These variables may affect the variation in PSC scores and could therefore have an impact on the results. The study included only two nursing homes and was conducted in only one municipality. Thus, generalizing should be done with caution. In addition, the results should be interpreted with caution when comparing the findings from this study to studies from other countries, as also stated in previous studies on PSC [36]. Differences between countries may be related to differences in how reporting of adverse events is treated and to variation in composition of the work force [36]. Because of the high number of comparisons between groups, there is also an increased risk of type I error and thus a possibility that some of the significant findings were just chance findings. Another limitation is that the questions about knowledge used in the study only capture the nursing personnel’s perceptions about knowledge and not the actual knowledge.

## Conclusions

Advanced education and familiarity with diabetes guidelines among nursing home personnel were associated with positive perceptions in key areas of PSC and may therefore play an important role concerning patient safety and quality of care. Further research is warranted to explore whether training programs to increase diabetes knowledge and promote the implementation of clinical guidelines in nursing homes can be related to the perception of PSC level.

## Abbreviations

NHSPSC: the Nursing Home Survey on Patient Safety Culture
PSC: Patient safety culture
